# Contrast-enhanced ultrasound evaluation of renal microcirculation in sheep

**DOI:** 10.1186/s40635-014-0033-y

**Published:** 2014-11-26

**Authors:** Antoine G Schneider, Paolo Calzavacca, Anthony Schelleman, Tim Huynh, Michael Bailey, Clive May, Rinaldo Bellomo

**Affiliations:** Intensive Care Unit, Austin Health, Austin Health, 145 Studley Road, Heidelberg, Victoria 3084 Australia; Australian and New Zealand Intensive Care Research Centre, School of Public Health and Preventive Medicine, Monash University, Commercial Rd., Melbourne, VIC 3004 Australia; Florey Institute of Neuroscience and Mental Health, University of Melbourne, Grattan Street, Parkville, VIC 3010 Australia; Radiology department, Austin Health, 145 Studley Road, Heidelberg, VIC 3084 Australia

**Keywords:** Renal perfusion, Microcirculation, Contrast-enhanced ultrasonography

## Abstract

**Background:**

Contrast-enhanced ultrasonography (CEUS) is a novel imaging modality to estimate microvascular perfusion. We aimed to assess renal cortical microcirculatory changes by CEUS during pharmacologically or mechanically induced modifications of renal blood flow (RBF) in experimental animals.

**Methods:**

We implanted invasive transit-time Doppler flow probes and a vascular occluder around the renal artery in six Merino sheep. After induction of general anaesthesia, renal CEUS studies with destruction-replenishment sequences were performed at baseline and after different interventions aimed at modifying RBF. First, we administered angiotensin II (AngII) to achieve a 25% (AngII 25%) and 50% (AngII 50%) decrease in RBF. Then, we applied mechanical occlusion of the renal artery until RBF decreased by 25% (Occl 25%) and 50% (Occl 50%) of the baseline. Finally, a single dose of 25 mg of captopril was administered. CEUS sequences were analysed offline with dedicated software and perfusion indices (PI) calculated.

**Results:**

Pharmacological reduction of RBF with AngII was associated with a 62% (range: 68 decrease to 167 increase) increase (AngII 25%) and a 5% increase in PI (range: 92% decrease to 53% increase) (AngII 50%) in PI. Mechanical occlusion of the renal artery was associated with a 2% (range: 43% decrease to 2% increase) decrease (Occl 25%) and a 67% (range: 63% decrease to a 120% increase) increase (Occl 50%) in PI. The administration of captopril was associated with a 8% (range: 25% decrease to a 101% increase) decrease in PI. Pooled changes in PI failed to reach statistical significance. The study was limited by the difficulty to obtain high quality images.

**Conclusions:**

CEUS-derived parameters were highly heterogeneous in this sheep model. The current protocol and model did not allow the evaluation of the correlation between macro and microcirculation assessment by CEUS.

## Background

Acute kidney injury (AKI) is a common and important complication of critical illness associated with increased morbidity, mortality and costs [1,2]. Alterations in renal perfusion are thought to play a central role in its pathogenesis. This causal relationship remains; however, largely speculative as data on renal perfusion in AKI and critical illness are scarce [3]. Moreover, methods enabling renal perfusion quantification are either invasive, very expensive, or difficult to apply in critically ill patients.

Contrast-enhanced ultrasonography (CEUS) is a recent imaging modality which provides a unique means of visualizing tissue perfusion. Several studies have suggested that CEUS could enable the quantification of blood flow in an organ [4,5]. These techniques have been used in the brain [6], myocardium [7] and, to some degree, in the kidney [8-10]. CEUS would be an ideal tool in the intensive care unit because it is safe [11] and applicable on the bedside [12]. Indeed, knowledge of renal perfusion changes after an intervention has the potential to greatly influence medical management in critical illness. However, challenges remain before renal perfusion quantification with CEUS can be used in clinical practice and further studies are required to validate its measurements.

In this study, we aimed to estimate renal cortical microcirculatory changes by CEUS during pharmacologically or mechanical induced modifications of renal blood flow (RBF).

## Methods

### Animal preparation

Experiments were conducted on six adult (1 to 2 years of age) Merino ewes. The experimental procedures were approved by the animal experimental ethics committee of the Florey Institute of Neuroscience under the guidelines laid down by the National Health and Medical Research Council of Australia.

All animals underwent two sterile procedures under general anaesthesia at 2 weeks intervals, and a similar 2 weeks recovery period was allowed before the experiment was undertaken. For all procedures, anaesthesia was induced with intravenous sodium thiopentone (10 to 15 mg/kg) for intubation followed by maintenance with oxygen/air/isoflurane (end-tidal isoflurane, 1.6% to 2.0%), peri-procedural antibiotic prophylaxis (procaine penicillin, Troy Laboratories Ptd Ltd, Smithfield, NSW, Australia or Mavlab, Qld, Australia) was administered and post-surgical analgesia maintained with intramuscular injection of flunixin meglumine (1 mg/kg) (Troy Laboratories or Mavlab, Qld, Australia) before and 24 h following surgery. Of note, the last exposure to NSAID was at least 2 weeks prior to the experiment.

During the first procedure, a carotid arterial loop was created to facilitate subsequent arterial cannulation and a transit-time flow probe (20 mm, Transonics Systems, Ithaca, NY, USA) was implanted in the pulmonary artery through left side thoracotomy. During the second procedure, a transit-time flow probe (4 mm) and a renal artery occluder (4 mm, IVM, In Vivo Metric, Healdsburg, California, USA) were placed around the left renal artery.

On the day before the experiment, cannulae were inserted into the carotid arterial loop for continuous arterial pressure monitoring and into a jugular vein for drugs infusions. Analogue signals (mean arterial pressure (MAP), heart rate, cardiac index (CI) and RBF) were collected on computer using a customized data-acquisition system (Spike2; CED; Cambridge, UK). The data were continuously recorded at 100 Hz and averaged every minute during experiments. To prevent major interferences with ultrasound equipment, RBF acquisition was interrupted during CEUS scans.

### Experimental protocol

To prevent pseudo-anaphylaxis and secondary pulmonary hypertension in response to ultrasound contrast agents (UCA) administration which was observed in other species [13,14], pre-medication with 0.5 mg dexamethasone (DBL®, Hospira, Wasserburg, Germany) per kg was administered 2 h before the administration of the UCA.

General anaesthesia was induced and maintained as described above. At least 15 min was allowed for the stabilisation of all parameters before RBF manipulations were started. Each intervention was preceded by 5 min of baseline haemodynamic data collection. After each intervention, a recovery period of at least 15 min was allowed.

Three interventions were performed to manipulate RBF. RBF was decreased using a continuous infusion of angiotensin II (AngII, Hypertensin Ciba™, Ciba-Geigy, Basel, Switzerland) with rates set to target a 25% (AngII low) and 50% (AngII high) decrease in RBF. Second, a mechanical decrease of RBF was achieved using the renal artery vascular occluder. The level of inflation of the occluding device was titrated to aim for a 25% (Occl 25%) and a 50% (Occl 50%) decrease in RBF as measured in real time by the transit-time flow probe. Finally, 25 mg of captopril (Captopril, E.R. Squibb & Sons, Princeton, New Jersey, USA) was given as a bolus with the intent to increase RBF. The two interventions aiming at decreasing RBF (AngII administration and occlusion) were performed in a random order by the investigator in charge of anaesthesia maintenance (PC). Captopril administration was always performed last.

At each study time, once hemodynamic parameters (RBF, MAP and CI) had stabilized, a CEUS scan of the left kidney (detailed procedure *infra*) was performed.

### CEUS procedure

For this study, we used Sonovue® (Bracco, Milano, Italy) as an UCA. The UCA was infused into a central vein using a dedicated syringe pump (VueJect®, Bracco Research, Geneva, Switzerland). Low mechanical index (MI = 0.06) ultrasound of the left kidney was performed with a Philips IU22® ultrasound machine (Philips, Amsterdam, The Netherlands) and a C5-1® 5 MHz probe. A long axis view of the kidney was obtained by placing the transducer probe over the lower back of the animal. Once adequate images of the kidney were obtained, UCA infusion was started at 1 ml/min. Image depth, focus, gain and frame rate were optimized at the beginning of each experiment and were held constant during the study. After a 2-min period required to obtain a steady state, five consecutive destruction/refilling sequences (with 15 s refilling time) were obtained [15,16]. Destruction was obtained by applying a flash of increased ultrasound intensity (5 pulses with high mechanical index (>1.0)).

### Sequence analyses

**Figure 1:**
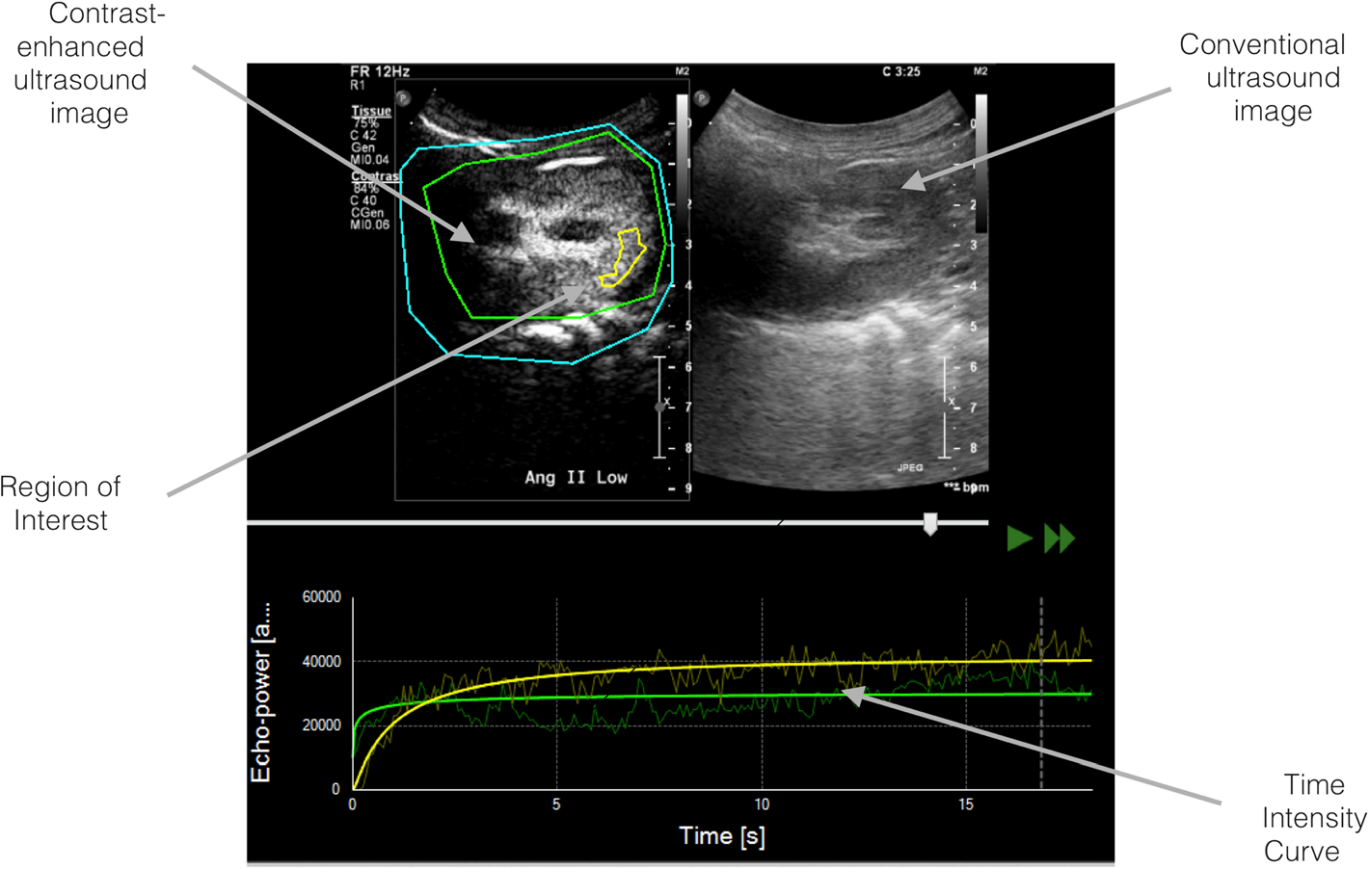
**Software analysis data.** Top left panel shows contrast-enhanced ultrasound images with region of interest drawn (yellow line). Top right panel shows conventional (B mode) imaging for anatomical localisation. Bottom panel shows linearized time intensity curve for the region of interest (yellow curve) and overall image (green curve_not relevant for perfusion quantification). AngII: angiotensine II.

Ultrasound data sets were exported in a digital imaging and communication in medicine (DICOM) format and analysed offline using VueBox® (Bracco Research, Geneva, Switzerland), a dedicated software package. An example of offline analysis is presented in Figure 1. Suboptimal sequences with inadequate contrast enhancement or excessive (or off plane) movement artefact were excluded. For each sequence, one region of interest (ROI) was drawn. In order to minimize the influence of local perfusion heterogeneities, this ROI was drawn so that it enclosed the largest area of visible renal cortex on the surface of the kidney closest to the ultrasound probe. When parametric map depicted high heterogeneity in the area, the ROI was adapted to exclude nonrepresentative areas. For instance, cortical areas that were only intermittently visible because of breathing or other artefacts and areas representing blood vessel transections were not included in the ROI.

The software generates linearized time intensity curves from which mean transit time (mTT) and relative blood volume (RBV) parameters are computed. These parameters have been described in detail elsewhere [17,15]. In brief, RBV is proportional to the local fractional blood volume as well as to the contrast agent concentration and is expressed in arbitrary units (AU). The mTT is a measure of time needed to replenish the imaging plane with fresh bubbles following destruction by ultrasonic flash and is inversely proportional to blood flow velocity; mTT is expressed in seconds. A perfusion index (PI) is obtained by calculating the ratio RBV/mTT. PIs are thought to be proportional to perfusion within a region of interest and are expressed in AU.

For each subject and study time, the median value for interpretable measurements was considered for analysis. Results from CEUS examination are reported as mean values and as percentage changes from the nearest baseline for RBV, mTT and PI. Given the expected inter-observation variability and based on previous research [8], a change of more than 25% between two measurements was considered to be significant.

### Repeat baseline measurement

As per study protocol, CEUS baseline measurements were obtained at the start of the experiment and were only repeated in case of significant modification of RBF or other hemodynamic parameter as compared with baseline values. Therefore, a baseline measurement was not repeated before each series of occlusions and before captopril administration. In such cases, and in cases where baseline measurement were discarded for poor quality, changes in CEUS-derived parameters were compared to the nearest baseline available.

### Statistical analysis

RBV, mTT and PI are reported as median (interquartile range). In addition, as baseline values are known to be highly heterogeneous due to inter-subject variability (organ depth, subcutaneous thickness and composition), we report changes in those values as median percentage change (range) from the nearest baseline, each animal being its own control. Analyses were performed using SPSS® version 21 (IBM, Armonk, NY, USA). All outcomes were assessed for normality and as RBV, mTT and PI were all well approximated by log-normal distributions, each was log-transformed prior to analysis. RBF measurements were found to be normally distributed. A two-sided *p* value of 0.05 was considered to be statistically significant.

## Results

### Renal blood flow

**Figure 2:**
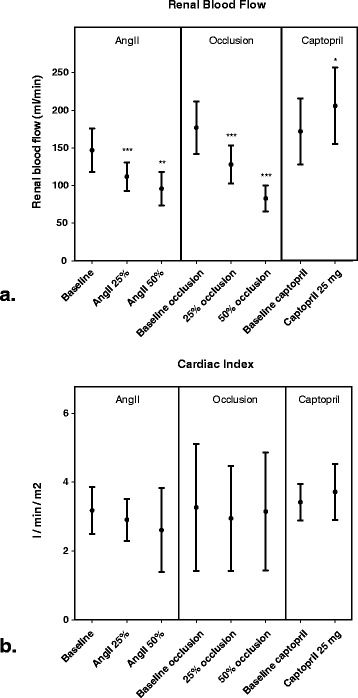
**Changes in hemodynamic parameters.** **(a)**. Renal blood flow as measured by implanted transit-time flow probes. **(b)**. Cardiac index as measured by pulmonary artery catheter. Presented values are means +/− standard deviation (error bars). *p value < 0.05. **p value < 0.01. ***p value < 0.001. AngII: angiotensine II. NB: 50% occlusion was obtained in 4/6 animals.

As shown in Figure 2, both pharmacological and mechanical interventions were associated with the expected, proportional and significant changes in renal blood flow (RBF).

Target reductions in RBF were obtained in all animals with AngII infusion. Overall, RBF decreased from a baseline of 147 (±29) to 112 (±19) ml/min (AngII low, *p* < 0.001) and to 96 (±22) ml/min (AngII high, *p* < 0.01).

Mechanical occlusion of the renal artery induced a 25% reduction in RBF in all animals and a 50% reduction in 4/6 animals. Overall, such occlusion was associated with a decrease in RBF, from a baseline of 177 (±34.9) to 128 (±25.5) ml/min (Occl 25%, *p* < 0.001) and to 83 (±17.2) ml/min (Occl 50%, *p* < 0.001). Finally, the administration of captopril was associated with an increase in RBF from 172 (±44) to 206 (±50.9) ml/min (*p* < 0.05).

### Pooled CEUS-derived parameters

**Figure 3:**
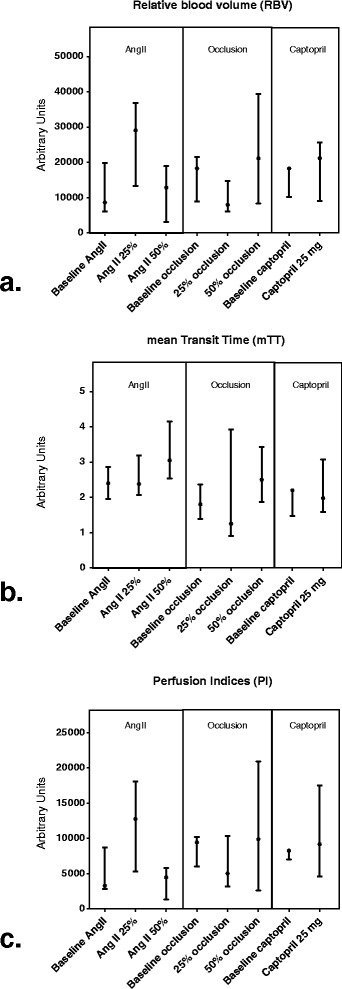
**Mean CEUS-derived parameters.** **(a)**. Relative blood volume (RBV), **(b)** mean transit time (mTT), **(c)**. perfusion indices (PI). Presented values are median and interquartile range (error bars) NB: interpretable baseline measurement available in 4/6 (occlusion and AngII) and 4/6 (captopril) animals. AngII: angiontensine II.

Median CEUS-derived parameters are reported in Figure 3.

Overall, most differences were found to be non-significant. The only two changes in CEUS-derived parameters which reached statistical significance were a decrease in RBV associated with a 25% reduction of RBF with mechanical occlusion and an increase in mTT associated with the high dose of AngII that caused a 50% reduction in RBF.

### Values expressed as percentage change from the nearest baseline

**Figure 4:**
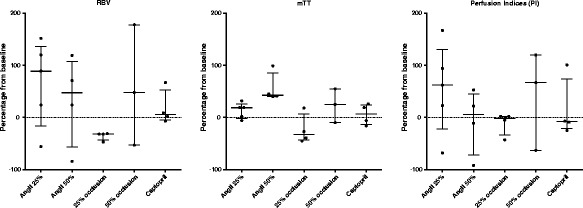
**Changes in perfusion indices expressed as percentage of the nearest baseline.** Each data point corresponds to one subject. Horizontal line bars correspond to median values and error bars to interquartile range.

Values of PI expressed as a percentage change from the nearest baseline (using each individual animal as its own control) are presented in Figure 4.

#### Angiotensin II infusion

A 25% reduction in RBF as induced by AngII infusion was associated with a median 89% increase in RBV (range: 56% decrease to a 152% increase) and a 19% median increase in mTT (range: 6% decrease to 32% increase) resulting in an overall 62% median increase (range: 68 decrease to a 167 increase) in PI.

A 50% reduction in RBF as induced by AngII infusion was associated with a median 47% increase in RBV (range: 84% decrease to 119% increase) and a 43% (range: 40 to 99%) increase in mTT resulting in an overall 5% increase in PI (range: 92% decrease to a 53% increase).

#### Mechanical occlusion

A 25% reduction in RBF as induced by occluder inflation was associated with a median 32% decrease in RBV (range: 31% to 47%) and a 33% decrease (range: 45% decrease to a 18% increase) in mTT resulting in an overall 2% decrease (range: 43% decrease to 2% increase) in PI.

A 50% reduction in RBF as induced by occluder inflation was associated with a median 48% increase in RBV (range: −53% decrease to a 178% increase) and a 25% increase in mTT (range: 10% decrease to a 55% increase) resulting in an overall 67% increase in PI (range: 63% decrease to a 120% increase).

#### Captopril

The administration of captopril was associated with a median 5% increase in RBV (range: 7% decrease to a 67% increase) and a 7% increase in mTT (range: 16% decrease to a 26% increase) resulting in an overall 8% decrease in PI (range: 25% decrease to a 101% increase).

### Data consistency

#### Missing values

Four baseline values were not repeated (as per study protocol) and a 50% reduction of RBF could not be obtained in two situations. In addition, CEUS data were judged as not interpretable at four time points (two baselines, one during AngII low dose infusion and one during 25% occlusion). Hence, altogether, valid results were obtained in 38/48 (79.9%) of study time points. Most (5/10) missing values occurred during the occlusion phase of the experiment.

#### In-between animals consistency

**Figure 5:**
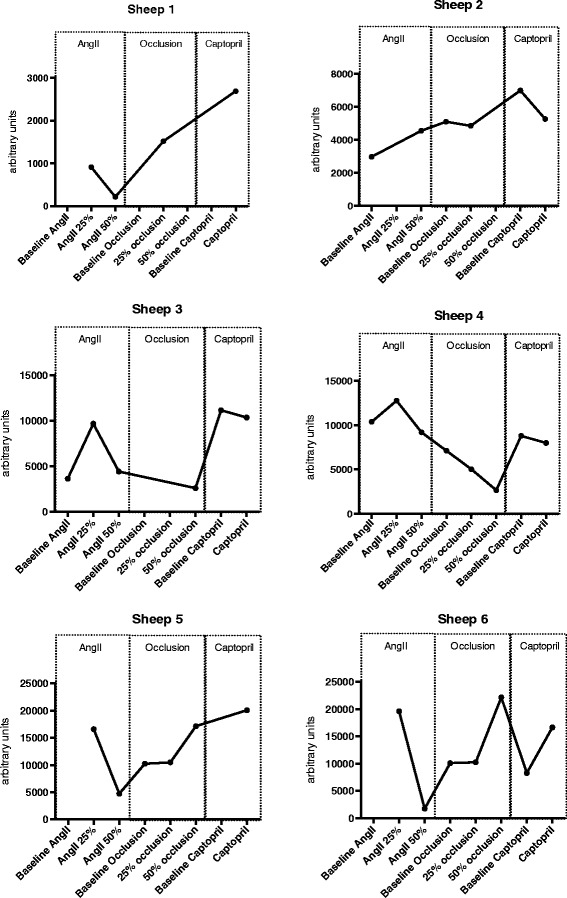
**Individual data (perfusion indices) for the six sheeps.** AngII: angiotensine II.

Individual animal data are presented in Figure 5. There was poor consistency for derived CEUS-PIs between animals and heterogeneous response to RBF manipulations.

## Discussion

### Summary of key findings

We performed an observational study in six merino sheep to evaluate changes in renal cortical microcirculation as evaluated by CEUS in response to pharmacological and mechanical modifications of RBF. We found that appropriate reductions in RBF could be obtained in all animals with an infusion of AngII and, in most of them, with an implanted renal artery occluder and that a 20% increase in RBF could be obtained with the administration of 25 mg of captopril. However, we found that obtaining high quality images in this sheep model was challenging. We also found that, within the limitations of our model, CEUS-derived perfusion indices did not parallel changes in RBF and that the findings were highly heterogeneous. We found that a similar decrease in RBF was associated with unpredictable and divergent changes in perfusions indices according to whether they were obtained with a mechanical occlusion of the artery or the administration of AngII.

### Comparison with previous studies

A previous human study has used CEUS to compare changes in renal microcirculation in response to changes in renal perfusion as induced by AngII and Captopril [8]. In this study, AngII administration was associated with marked and statistically significant decreases in PI, even when renal plasma flow was only reduced by 15%.

A recent animal study [10] used CEUS to measure renal microcirculation parameters in rats. Similar to our protocol, the investigators used AngII to decrease RBF. This study compared two methods of blood flow quantification and demonstrated an excellent correlation between CEUS-derived parameters and blood flow. We failed to replicate these data in our sheep model. This might in part be due in part to our decision to use parasagittal images, as the findings from the above study suggested that coronal images had better sensitivity.

In addition, our results were associated with important heterogeneity. Heterogeneity among subject and measurements has been previously reported, however to a much lesser degree than in the current study. Potential reasons for increased heterogeneity might involve the model used and our study design and are discussed in the ‘Strengths and Limitations’ section.

A moderate (25%) reduction in RBF as induced by AngII was associated with an increase in PI, while a larger (50%) reduction was associated with a decrease in PI. This was essentially linked to a proportional increase in mTT (indicative of delayed replenishment) which reached statistical significance during 50% occlusion associated with a persistent increase in RBV (indicative of increased UCA concentration).

These findings seemed quite robust as such pattern was exhibited in similar magnitude in all but one animal where a baseline was available. This could be explained by the specific effect of AngII on renal microcirculation. Indeed, both afferent (AA) and efferent (EA) arteries respond to AngII with a dose-dependent vasoconstriction [18]. Such response is significantly more pronounced at the EA level with vasoconstriction occurring at lower concentration [19,20]. Therefore, a proportional increase in transit time with increased AngII induced RBF reduction could be expected. The associated increase in UCA concentration (RBV) remains to be investigated and might well be artefactual (see ‘Strengths and Limitations’ section).

To the best of our knowledge, no study has evaluated changes in renal cortical microcirculation in response to renal artery occlusion. The influence of a mechanical occlusion on renal function was described using a similar model of sheep [21]. In this study, neither an acute (30 min) 25%, 50% or 75% nor a prolonged (80% for 2 h) reduction in RBF as induced by an implanted occluder was associated with sustained loss of kidney function. In this study, occlusion phases were associated with transient increases in MAP with returns to baseline within 2 h.

In comparison, we found that a similar decrease in RBF was associated with a modest (+11% with Occl 25%) or marked (+41% Occl 50%) *increase* in renal cortical microcirculation. These changes were associated with no (Occl 25%) or a modest (+14 mmHg, Occl50%) increase in mean arterial pressure but no change in cardiac output. This could be consistent with modest activation of the renin-angiotensin-aldosterone system (RAS) [22,23]. Hence, such progressive activation might explain the paradoxical increase in perfusion indices in the same fashion as observed with low dose AngII (predominant EA vasoconstriction). However, given the small number of observations such conclusions remain speculative.

### Strengths and limitations

Heterogeneity of measurements is a common limitation of all attempts to use CEUS for organ perfusion quantification. Sources for this heterogeneity have been reviewed in details by Tang et al. [24]. In order to limit the influence of the most important factors associated with heterogeneity, we kept mechanical index, dynamic range, frequency and gain constant throughout the experiment.

Ensuring the reproducibility of anatomical location of ROI proved challenging in our experimental animals. For adequate perfusion quantification, focus depth and insonification angle need to be kept constant. In sheeps, kidney position seems to be greatly altered by respiration and peristaltic activity. To overcome this limitation, we have used anatomical landmarks and compared live images with previously acquired baseline images. In addition, several sequences were obtained at each study point and only similar looking areas were retained for data analyses. Finally, we tried to locate ROIs at similar depth and distance from the focal depth to ensure a more uniform acoustic field as recommended by Averkiou et al. [25]. Motion artefact was partially tackled by the selection of a probe angle limiting this motion and by the use of an advanced image stabilisation algorithm in the VueBox® software. Unfortunately, such compensation can only deal with in-plane motion and perhaps further studies should make use of 3D probes [26]. Further attenuation of signal was associated with transient interposition of air fluid cavities possibly related to the sheep's large four-compartment stomach. This issue limited the number of usable sequences.

Further heterogeneity might have been due to protocol-related changes in blood pressure. Indeed, such changes are known to directly to affect the mean size of the bubbles and their resonant frequency. Mor-Avi et al. [27] observed an approximately 20% decrease in video intensity for albumin-coated bubbles during systole compared with diastole. However, changes in blood pressure observed in our protocol are of lesser intensity, and their influence on the results might be limited.

Changes in the inhaled fraction of anaesthetic gas might have impacted the intensity of CEUS signal [28]. Indeed, inhaled gas concentrations might alter microbubble's size and dynamically change their response to the ultrasound beam and be associated with a decrease in their blood concentration as larger bubbles tend to be filtered in the lung. To overcome this limitation, only minor and strictly mandatory changes in inspired fractions of gases were allowed during the experiment. In addition, anaesthetic gas might have decreased RBF through its effects on the activation of the RAS [29]. However, this is unlikely to have influenced our results as study interventions were targeted based on the readings of a flow meter.

The increase in intensity sometimes observed during the second administration of contrast agent in the same subject has been related to the saturation of pulmonary macrophages by the first injection, leading to increased signals from the second injection [24]. The importance of this effect in sheep and its impact on our results is unknown.

The administration of dexamethasone as a pre-medication to prevent pseudo-anaphylaxis was necessary. However, such medication might influence the circulation and perhaps explain the shift in RBF seen throughout the study. However, as repeated baseline measurements were taken, such shift is unlikely to have biased our results.

Finally, our study protocol might not have allowed enough time between interventions to enable establishment of a new steady state in the microcirculation. This is suggested by the fact that the ‘baseline’ value for RBF did not systematically return to their ‘original’ baseline and presented a trend to an upward shift. This limitation was partially attenuated by the repetition of baseline measurements. Unfortunately, such measurements were not performed before each series of occlusions.

### Implications for clinicians and further studies

Renal perfusion quantification with CEUS remains a promising yet non-validated tool.

Indeed, the lack of a gold standard for the quantification of microcirculatory perfusion and the large heterogeneity of the results (and its associated potential for major error) limits the application of this technology in a clinical setting for the time being.

Most of the aforementioned sources of variability are likely to primarily influence the intensity of the enhancement by the UCA, therefore the RBV parameter is that most likely to be associated with measurement errors.

Further studies are required to improve technical measurements in order to limit heterogeneity and better validate this technique. These studies should aim at determining ideal conditions to limit measurements heterogeneity.

## Conclusions

CEUS-derived parameters were highly heterogeneous in this sheep model. The current protocol and model did not allow the evaluation of the correlation between macro- and microcirculation assessments by CEUS. Further studies should include other animal models and simpler protocols and possibly use of a 3-D probe.

## Abbreviations

AA: afferent artery
AKI: acute kidney injury
AngII: angiotensin II
AU: arbitrary units
CEUS: contrast-enhanced ultrasonography
EA: afferent artery
MAP: mean arterial pressure
mTT: mean transit time
PI: perfusion index
RAS: renin-angiotensin-aldosterone system
RBF: renal blood flow
RBV: relative blood volume
ROI: region of interest
UCA: ultrasound contrast agent
